# Combined laparoscopic and posterior approach resection of large sacrococcygeal cystic teratoma

**DOI:** 10.1186/s40792-020-01104-4

**Published:** 2021-01-13

**Authors:** Ziyad Alyousef, Maryam Aleissa, Ohood Alaamer, Nahar Alselaim

**Affiliations:** 1 College of Medicine, King Saud Bin Abdulaziz University for Health Sciences, Riyadh, Saudi Arabia; 2 Department of Surgery, King Abdulaziz Medical City, Riyadh, Saudi Arabia; 3 Prince Noura Bin Abdulrahman University, Riyadh, Saudi Arabia; 4 King Abdullah International Medical Research Center, Riyadh, Saudi Arabia; 5 Department of Surgery, King Faisal Specialist Hospital & Research Center, , Riyadh, Saudi Arabia

**Keywords:** Retrorectal mass, Sacrococcygeal teratoma, Combined laparoscopic approach, Sacrococcygeal mass

## Abstract

**Background:**

Teratoma is a true neoplasm and originates from the three germ cell layers and it can contain any tissue derived from these layers. The location of teratoma is variable according to the age group. In adults, sacrococcygeal teratoma is rare and carries a low risk of malignant transformation. Surgical resection is the mainstay of treatment and is challenging due to tumor location.

**Case presentation:**

We are presenting a case report of a 16-year old female referred to our hospital with recurrent attacks of urine retention. Imaging study showed a large sacrococcygeal tumor. It was successfully resected by a combined laparoscopic and posterior approach without any major complication.

**Conclusion:**

The combined laparoscopic and posterior approach is a safe surgical technique for resection of the large sacrococcygeal tumor. This surgical method has been published around 10 times in separated reports around the world and for first time in our region.

## Introduction

Teratomas are tumors which contain tissues resembling normal derivatives from all the three germ cell layers. These tumors could be benign or malignant, most commonly found in testes and ovaries but also found in retroperitoneum, mediastinum, sacrococcygeal region and intracranial spaces [1].

The sacrococcygeal teratoma is commonly found in the neonate with an incidence of 1/40,000 live births [2, 3]. The sacrococcygeal/Retrorectal location is the commonest location for teratomas in infants [2], and is associated with anorectal, vertebral, urinary tract, musculoskeletal and cardiac congenital anomalies in about 15% of neonatal tumors [2, 4, 5]. In infants 90% of these tumors are externally visible whereas in adults most are intrapelvic, with 75–90% of all cases occur in females [3, 6].

Presacral tumors present later than tumors with an external component and are associated with a higher rate of malignant change [7].

In this case report, we are presenting our experience of removing a large sacrococcygeal tumor by a combined laparoscopic and posterior open approach.

## Case presentation

**Fig. 1:**
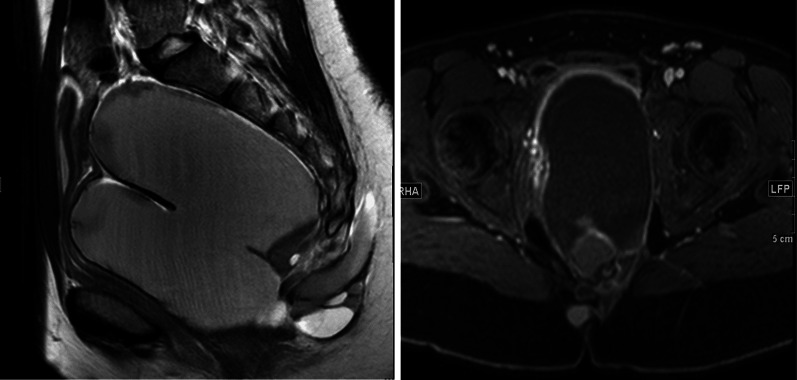
Large multiloculated pre-sacral pelvic cystic lesion with small Sacrococcygeal extension causing mass effect,displacing the rectum to right side

A 16-year-old Saudi female had multiple emergency department visits for new acute onset of recurrent episodes of urinary retention over few weeks requiring intermittent bladder catheterization. Her workup showed a pelvic mass and was referred to the authors’ outpatient clinic for management mid of 2019. History and physical examination were unremarkable with BMI of 24.6. Pelvic Magnetic resonance imaging (MRI) showed a large multiloculated cystic presacral tumor approximately measuring 11.5 × 8.8 × 12.3 cm. A small part of the tumor was seen crossing the left levator ani muscle into the left ischioanal fossa and insinuating posterior to the last coccygeal joint with no bone destruction or intraspinal extension, Altman type IV. The lesion contained multiple cystic locules some containing mucoid material while others contained hemorrhage. The tumor was displacing the rectum to the right side, the uterus and the bladder anteriorly and stretching the vagina (Fig. 1). Her workup was completed with tumor markers which were negative, colonoscopy with no other tumors, and Positron emission tomography (PET) scan with no worrisome uptake. The perioperative impression was a benign lesion, however, surgical resection advised to relive the patient’s symptoms, the risks and benefits of surgery were discussed with the patient and her parents who consented for surgical resection.

## Surgical technique

**Fig. 2:**
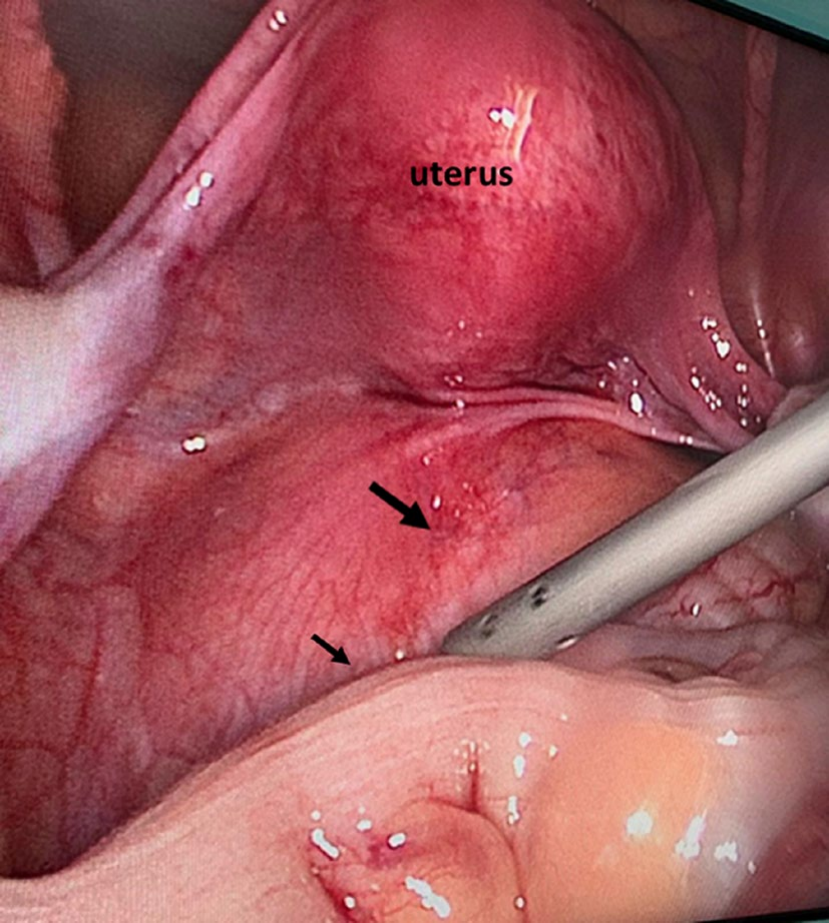
Intra-operative fining of Sacrococcygeal mass (long arrow) pushing the rectum (short arrow) anteriorly to the right side

**Fig. 3:**
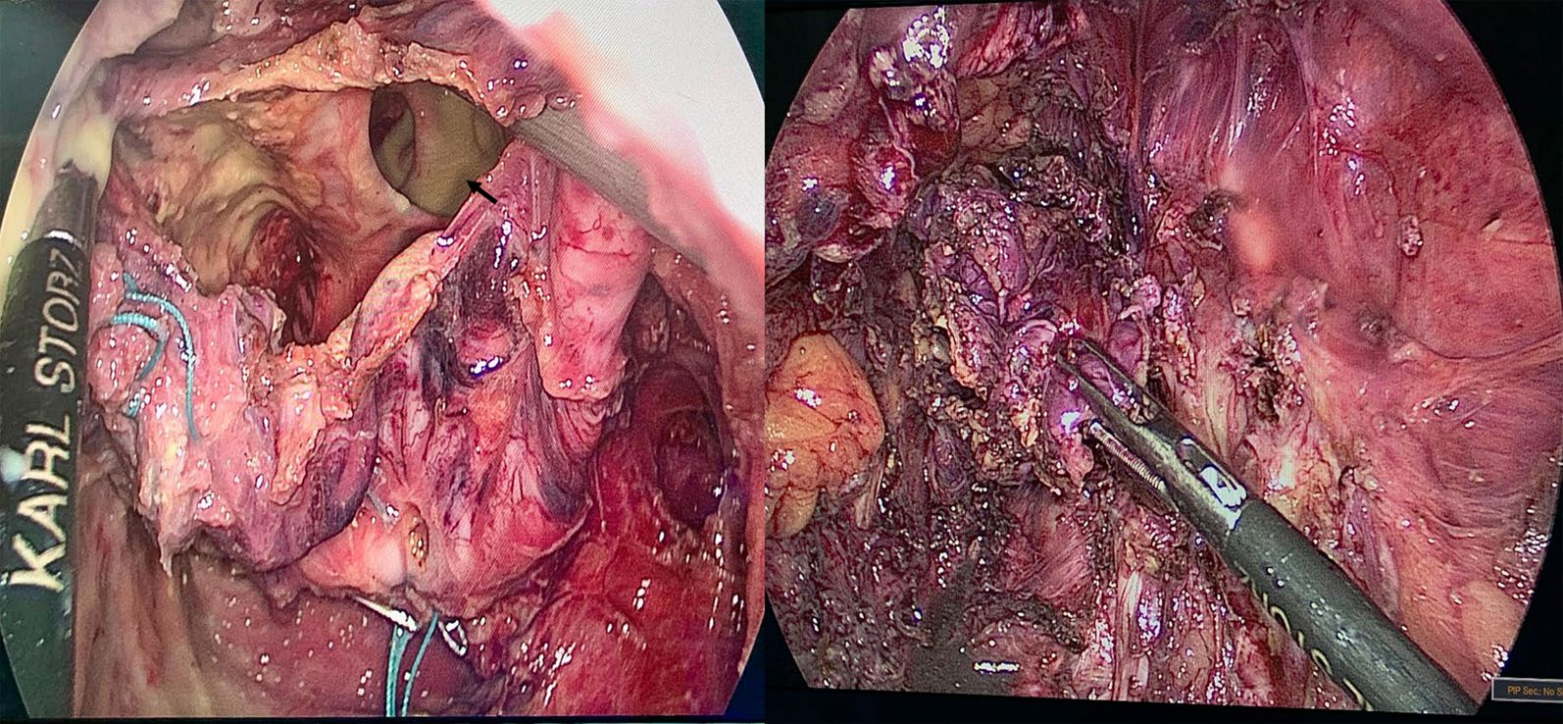
Intraoperative finding of yellow colour material spill from the cystic tumour once it was open to facilitate its dissection

The patient had a mechanical bowel preparation one day before surgery. General anesthesia was given with endotracheal intubation and the patient was placed in a lithotomy position. Her abdomen and perineum were prepped and draped. An umbilical 11 mm balloon trocar was passed by open technique and the camera was inserted. Additional 3 ports were used in the surgery, two 5 mm trocars were passed on the right of umbilicus for surgeon and one on the left side of umbilicus for retraction. The tumor was in the retrorectal space pushing the rectum anteriorly and to the right side (Fig. 2). Dissection was started using a harmonic dissector in the plane between the mass and the rectum after identifying the left ureter. The dissection was done all around the mass and deep into the pelvis. The mass got accidentally opened and thick yellow fluid came out which was carefully suctioned, there was no bleeding inside the mass, in contrast to MRI report (Fig. 3). The mass was dissected till we reached rectococcygeal ligament which was cut to reach the pelvic floor.

The remaining mass was extending posteriorly to the coccyx according to the imaging and it was decided to remove it through a posterior approach. A leak test showed a pinhole perforation on the left lateral side of the mid rectum, it was repaired with interrupted PDS 3/0 sutures. A repeat leak test was negative for any air leak from the repaired area. Since there was spillage, we elect to do copious irrigation with 5 L of normal saline fluid before hemostasis was secured. The pelvis was drained with a 19 French drain. The trocars were removed under vision, pneumoperitoneum released, and umbilical fascia repaired.

**Fig. 4:**
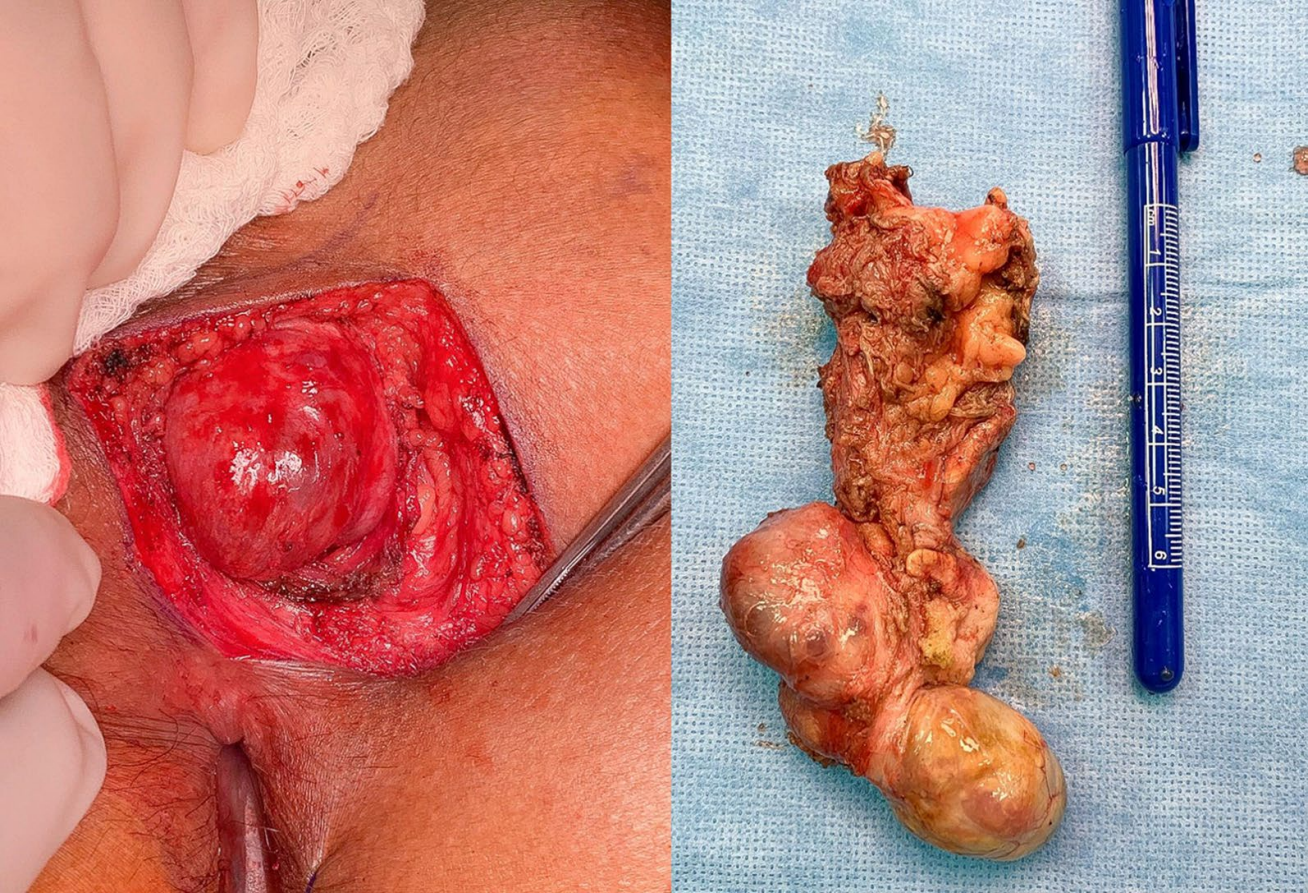
Paracoccygeal skin incision to access to the presacral space. The mass was identified and dissected carefully of the surrounding tissues and removed in single piece

The patient was placed in a prone position and a left para coccygeal incision extending to the posterior midline was made. Dissection was carried through the subcutaneous tissue and coccygeal ligament was transected to get access to the presacral space. The mass was dissected and excised carefully from the surrounding tissues (Fig. 4). The incision was closed in layers with one drain placed in the pelvis and other drain in subcutaneous tissues for seroma.

The postoperative course was uneventful. Computerized axial tomogram (CT) scan of the pelvis with rectal contrast showed no leak. The drains were removed, and she was discharged home after 15 days.

**Fig. 5:**
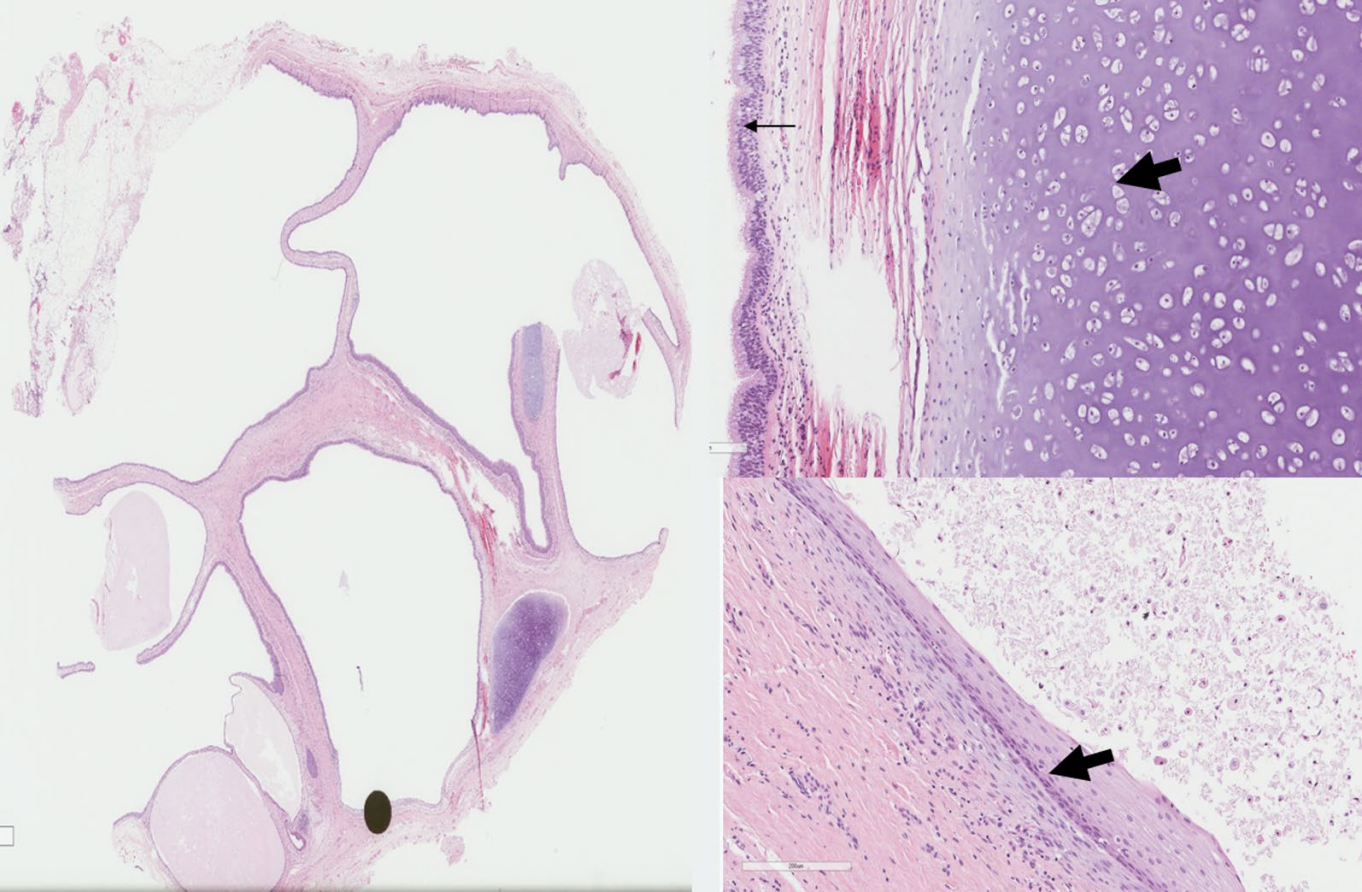
H&E slide from the lesion showing cystic structure with mixed histological. **a** Low power magnification shows multicystic lesion lined by epithelium tissue. **b** Higher power magnification shows cartilage (thick arrow) lined by respiratory type epithelium (thin arrow). **c** Higher power magnification shows cystic structure lined by squamous epithelium( thick arrow)

The histopathology showed a mature cystic teratoma measuring around 11 cm (Fig. 5).

The patient is following in the out-patient clinic with no urinary symptoms. CT scan done at one year show no evidence of recurrence.

## Discussion

In Adults, sacrococcygeal teratoma is rare [3]. These tumors may be asymptomatic or present with a combination of pelvic pain and/or palpable mass. Pelvic/lumbar/referred sciatic type pain may be associated with benign or malignant tumors but is more ominous for malignancy [1, 8]. There may be tumor-specific symptoms due to the local invasion of pelvic structures or symptoms like urine retention or rectal obstruction caused by pressure effect of an enlarging mass in the pelvis similar to the patient in this case report who was experiencing recurrent urinary retention without urinary tract infection due to a sacrococcygeal tumor [1].

Many patients complained of pain on sitting. The diagnosis of a presacral mass can usually be made with a digital rectal examination [6].

In the pediatric population with sacrococcygeal teratomas, there is a tendency toward malignant change with increasing age. In adults, however, benign tumors are more common. Presacral tumors present later than tumors with an external component and are associated with a higher rate of malignant change [7].

The standard of care for benign tumors is complete resection with negative margins, to relieve the symptoms and prevent malignancy in the future [8]. The retrorectal space is very narrow and contains vital structures that must be saved and this adds to the surgical difficulty.

Due to non-specific symptoms, the diagnosis is often made by imaging studies like CT, MRI and Ultrasound. Both CT and MRI had a 100% sensitivity in diagnosing a retrorectal tumor but imaging features alone cannot differentiate between benign and malignant teratomas [7], [8]. MRI is helpful in planning surgical resection of tumor by showing spinal canal invasion by MRI, [5] intrapelvic extensions and relationship to surrounding structures [7].

CT scan is very sensitive in showing calcification in more than 50% of malignant tumors, but other studies report calcification on CT in both benign and malignant teratomas [5, 7]. Hence tumor calcification cannot be used as a criterion to differentiate between benign and malignant tumors [1].

CT and MRI are excellent investigations defining the extent of sacrococcygeal teratomas, while MRI is better in detection of spinal canal invasion [5]. They also tell about the nature of mass whether solid, cystic or mixed, its intrapelvic extension and relationship to the surrounding structures. This information is vital for planning the surgery [7].

Sagar et al. showed the ability of MRI to differentiate between benign and malignant tumors with specificity and sensitivity of 97% and 88% respectively. The corresponding values for preoperative biopsy were 100% and 83% [9]. A biopsy of a retrorectal tumor should be avoided as it can cause possible contamination, tumor spread, abscesses, fecal fistula and meningitis. Biopsy is only justified in inoperable tumors to provide tissue diagnosis to guide adjuvant therapy [10].

The serum levels of alpha-fetoprotein (AFP), beta human chorionic gonadotrophin (BHCG) and carbohydrate antigen 19-9 (CA 19-9) can be raised in malignant teratomas. In our patient tumor markers were normal, most likely suggesting a benign teratoma [11].

The cornerstone of surgical management for retrorectal tumors is complete surgical resection [10, 12]. In general abdominal approach is used for tumors above the third sacral vertebra S3 while a posterior approach is used for tumors below S3 [1] and those involving nerves [12]. The major disadvantage of posterior approach is intraoperative hemorrhage and potential injury to lateral pelvic nerves [12].

A combined anterior and posterior approach was used for large tumors that could not be removed through a single incision [10]. Similarly in our patient a combined anterior laparoscopic and posterior open approach was used because of large size of the tumor.

The largest comprehensive review of surgical management of retrorectal tumors was published by Baek which had 1708 patients with 155 having teratoma. The overall postoperative complications in the open surgical approach were 13.2%. The morbidity associated with the posterior approach was 7.2% with anterior approach was 19.3% and with a combined approach it was 24.7% [12].

Post-operative complications included neurologic bladder 15%, wound infection 11%, dysesthesia 7%, fecal incontinence 7% massive bleeding 4% ureteric injury extensive soft tissue infection, reoperation for bleeding and impotence [12, 13].

The minimally invasive approach by laparoscopic surgery was proposed for the first time in 1995 by Sharpe as laparoscopy provided bright illumination and magnification for deep pelvic tumors posterior to the rectum and helped in better dissection avoiding trauma to rectum pelvic muscle, nerves and control of blood vessels especially the median sacral artery [14, 15].

In one large systemic review, minimally invasive approach (MIS) was used in 83 patients. The hospital stay was shorter than open surgery (4 ± 2 vs 9 ± 7 days *p* < 0.05). Complication rate was 19.8% in MIS and 12.2% in open surgery but was not statistically significant [12].

The use of laparoscopic approach provided advantages like improved visualization in the narrow pelvis which facilitated the dissection of the cystic lesion from the rectum, avoiding a large incision, less postoperative pain, and wound infections.

## Conclusion

After comprehensive preoperative planning large sacrococcygeal teratoma extending from the sacral promontory to beyond coccyx can be safely removed by a combined laparoscopic and posterior approach.

## Data Availability

Not applicable.

## Abbreviations

AFP: Alpha-fetoprotein.
BHCG: Beta human chorionic gonadotrophin
CA 19-9: Carbohydrate antigen 19-9
CT: Computerized axial tomogram
MRI: Magnetic resonance imaging
PET: Positron emission tomography
